# Implications of Metabolic Bariatric Surgery on Reproductive Health

**DOI:** 10.3390/jcm14155446

**Published:** 2025-08-02

**Authors:** Amihai Rottenstreich, Yitka Graham

**Affiliations:** 1 Department of Obstetrics and Gynecology, E. Wolfson Medical Center, Holon 5822012, Israel; 2 Gray Faculty of Medical and Health Sciences, Tel Aviv University, Tel Aviv 6997801, Israel; 3 Division of Maternal-Fetal Medicine, Department of Obstetrics and Gynecology, Zucker School of Medicine at Hofstra/Northwell, New York, NY 11549, USA; 4 Laboratory of Blood and Vascular Biology, Rockefeller University, New York, NY 10065, USA; 5 Helen McArdle Nursing and Care Research Institute, University of Sunderland, Sunderland SR1 3SD, UK; yitkagraham@gmail.com; 6 Surgical Weight Management Unit, Department of General Surgery, South Tyneside and Sunderland NHS Foundation Trust, Sunderland SR4 7TP, UK; 7 Faculty of Psychology, University of Anahuac, Mexico City 52786, Mexico; 8 Faculty of Biomedical Sciences, Austral University, Buenos Aires B1406, Argentina

**Keywords:** metabolic and bariatric surgery, reproductive health, family planning, contraception, pregnancy

## Abstract

In the last two decades, metabolic and bariatric surgery (MBS) has become the mainstay of treatment for severe and complex obesity. The majority of patients undergoing MBS are women of childbearing age. Coupled with the dramatic increase in the utilization of MBS, caregivers are likely to encounter patients who have undergone MBS in routine practice. From this perspective, we highlight the different reproductive health challenges and issues encountered throughout the pre-operative, peri-operative, and postoperative phases.

## 1. Introduction

Obesity is one of the foremost public health challenges of the twenty-first century. Its worldwide prevalence has nearly tripled in the last four decades, with the American and European regions having the highest rates [1,2]. Metabolic and bariatric surgery (MBS) has become the most effective treatment for severe and complex obesity, due to its established efficacy in achieving significant weight loss and improving obesity-related health complications and quality of life [3,4,5].

Up to eighty percent of persons who undergo MBS are women of childbearing age [3,6,7]. Furthermore, fertility in women with obesity generally improves after MBS, as menstrual irregularities and ovulatory problems tend to resolve after weight loss [8,9,10]. Additionally, healthcare providers are increasingly likely to encounter reproductive-aged women who have undergone MBS in their routine practice due to the increased utilization of MBS in the management of obesity [11,12].

**Figure 1:**
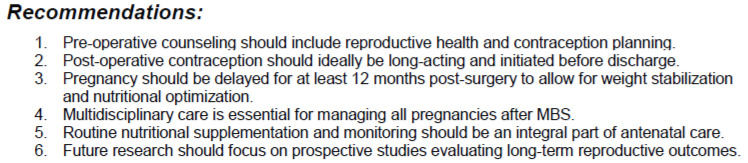
Key list of recommendations for reproductive-aged women undergoing metabolic and bariatric surgery.

MBS introduces unique challenges concerning women’s reproductive health throughout the pre-operative, peri-operative, and post-operative phases. In this article, we aim to provide guidance for healthcare professionals who care for these patients. A key list of recommendations is provided in Figure 1.

## 2. Pre-Operative Phase

In female patients undergoing MBS, appropriate reproductive and contraceptive counseling is critical. Moreover, a desire to improve fertility is an occasional motivation to undergo MBS in a substantial proportion of patients [13,14]. In a previous cross-sectional study from our group, most MBS surgeons have acknowledged the paramount importance of such conversations, and considered the preoperative stage to be the best time for initiating these discussions [13,15]. Nevertheless, while the majority of surgeons believe reproductive health discussions are important, they often feel uncomfortable and do not routinely initiate them [13,15,16]. This is evident by the relatively high proportions of MBS patients reporting the lack of such counseling, and many others who felt the discussions were insufficient [14,16]. One promising way to tackle this issue is to include reproductive-health counseling as part of the pre-operative checklist, ideally in a multidisciplinary fashion, thereby providing patients with comprehensive information at this stage [13,15,17]. The topics covered in this setting should include: sexual activity and contraception, future pregnancy and its timing, expected pregnancy outcomes, and the effect of pregnancy itself on long-term MBS-related outcomes. Such a checklist should be developed in collaboration with MBS multidisciplinary teams and sexual and reproductive health professionals so both patients and providers benefit from the collective expertise.

Nutritional deficiencies are one of the main challenges encountered in the postoperative management after MBS [18]. Due to their longer expected life span and the potential for future pregnancies, women of reproductive age are at increased risk of developing such deficiencies. We have shown that the presence of pre-operative anemia was the only independent risk factor for experiencing anemia during pregnancy [19]. Moreover, in those who plan to conceive after surgery, a higher dose of folic acid (i.e., 5 mg) is recommended in order to reduce the risk of neural tube defects in the offspring [17]. As such, proper nutritional evaluation and treatment as necessary is vital at the preoperative stage.

The practice guidelines cosponsored by the American Society for Metabolic and Bariatric Surgery, the Obesity Society, and the American Association of Clinical Endocrinology recommend discontinuing estrogen therapy one month prior to the procedure due to the postoperative risk of venous thromboembolism (VTE) [20,21]. Therefore, caregivers should actively screen MBS candidates preoperatively regarding contraceptive use. However, we have found that most surgeons do not routinely assess contraceptive use or guide their patients to discontinue estrogen-containing contraceptives [13] (Table 1).

## 3. Peri-Operative Phase

While the recommended surgery-to-conception interval is controversial, as will be discussed below, it is crucial to avoid the possibility of early pregnancy at the time of MBS, which could have hazardous implications [22]. This may be even more relevant in women who discontinued estrogen-containing contraception and did not initiate an alternative mode of contraception. While in some regions of the world routine pre-operative b-HCG testing is performed prior to any surgery among reproductive-aged women, in many other countries this is not routinely done. We support b-HCG testing prior to MBS procedures, and postponing the procedure if positive.

Sexual activity can be safely resumed at the perioperative period, provided that safe and effective contraception is utilized in order to prevent unintended pregnancy. Proper contraceptive counseling and provision should be an integral part of the management of reproductive-age patients undergoing MBS. The choice of contraception should be made through shared decision-making, considering individual health status, surgical factors, and patient preferences, and the expertise of reproductive health professionals should be sought. Any mode of estrogen-containing contraception should not be used in the early months after MBS due to the associated thromboembolic risk. Moreover, the physiologic and anatomic changes, especially with malabsorptive procedures such as Roux-en-Y gastric bypass and one anastomosis gastric bypass (OAGB), that follow MBS may increase the risk of oral-contraceptive failure. The trend toward decreased dosages of hormones in orally administered contraception might further place post-MBS patients at higher risk of unintended pregnancy. The U.S. Medical Eligibility Criteria (MEC) for contraceptive use advocate avoiding the use of both combined and progestin-only oral contraceptives after malabsorptive MBS procedures [23]. There are limited data on the utilization of oral contraceptives after sleeve gastrectomy. Nevertheless, it was shown that the reduction in stomach size following sleeve gastrectomy leads to decreased surface area and dilution volume, contributing to impaired drug bioavailability [24,25,26]. We also encountered relatively high rates of unintended pregnancy in patients using oral contraception after sleeve gastrectomy [27]. In our practice, in line with other groups, we remain cautious regarding the use of oral contraceptives after sleeve gastrectomy [15,28].

## 4. Post-Operative Phase

Menstrual pattern may be changed after surgery. In a cross-sectional performed among 1030 post-sleeve gastrectomy female patients, we have identified a significant improvement in the duration and regularity of menses, with a reduction in the incidence of prolonged (>7 days) menstrual bleeding [27], which concurs with additional reports [29,30,31,32,33]. This positive impact of MBS on menstrual cycle pattern is encouraging as abnormal uterine bleeding is common in patients with obesity with a direct negative influence on quality of life [34,35,36].

Relatively high infertility rates are found in MBS candidates [16,27]. In addition, as aforementioned, conceiving after surgery is often a motivation to undergo MBS [17]. MBS has been consistently shown to have a positive impact on ovulation and fertility [37,38]. MBS can therefore enhance the prospects of conceiving, including spontaneously, in infertile patients with obesity.

### 4.1. Pregnancy After MBS

Counseling in female patients of childbearing age undergoing MBS should include information regarding pregnancy and the expected maternal and perinatal outcomes. In a cross-sectional study from our group including post-MBS patients who conceived after surgery, most patients reported having inadequate information regarding the effects of MBS on pregnancy outcomes, further underscoring the need to improve counseling [14]. Pregnancy after MBS poses unique issues and challenges that will be discussed in this section.

#### 4.1.1. Timing of Pregnancy After MBS

Conceiving in the early post-operative period is advocated against by different professional society guidelines. Guidelines cosponsored by the American Society for Metabolic and Bariatric Surgery, the Obesity Society, and the American Association of Clinical Endocrinology [20], as well as those of the British Obesity and Metabolic Surgery Society and the American College of Obstetricians and Gynecologists [39,40], recommend delaying pregnancy for at least 12 months after surgery. The rationale behind these restrictions is to ensure that pregnancy does not occur during the rapid catabolic weight loss period, which may lead fetal malnutrition and impaired growth. As will be discussed below, higher rates of small-for-gestational-age (SGA) infants have been reported after MBS. However, data to support the association between surgery-to-conception interval and the delivery of an SGA infant remain sparse, with conflicting results reported [41,42,43,44,45,46]. We have shown that following sleeve gastrectomy, conceiving within 6 months after surgery was associated with the delivery of an SGA infant, with no association found for those who conceived within 6–12 months after surgery [47]. In our practice, we support the recommendations of the Royal College of Obstetricians and Gynaecologists [48] and other publications [49,50], suggesting that a more personalized approach should be adopted instead of the arbitrary fixed-time limitations. This is of particularly importance for older women toward to the end of their reproductive age or those who face fertility issues, for whom delaying pregnancy may compromise their chance of conceiving. These factors, coupled with the type of surgery and stabilization of weight loss [50], should be taken into account in the shared decision-making process regarding the optimal timing of pregnancy after surgery.

#### 4.1.2. Maternal and Perinatal Outcomes

MBS was shown to have different positive influences on pregnancy outcomes including a reduction in the rates of gestational hypertensive disorders and fetal macrosomia [43,51,52]. The effect on preterm birth rates was inconsistent [43,51,52].

Increased rates of SGA infants have been consistently reported after MBS [43,51,52,53,54]. Nevertheless, the risk factors associated with the delivery of an SGA infant are largely unclear. As aforementioned, short surgery-to-conception interval may be a potential risk factor. Higher rates of SGA infants have been reported after malabsorptive procedures compared to restrictive procedures [54], although an increased risk was also shown after sleeve gastrectomy [53,55]. Maintaining glycemic balance in order to avoid hypoglycemia is also of utmost importance to optimize fetal growth [56,57,58,59].

#### 4.1.3. Gestational Diabetes Screening

Gestational diabetes mellitus (GDM) screening in the general population is either performed using a two-step approach, in which a 50 g glucose challenge test (GCT) is followed (if abnormal) by a 100 g oral glucose tolerance test (OGTT). In other countries, a one-step approach includes the performance of a 75 g OGTT. Specific guidelines have not been established for this purpose in post-MBS pregnant patients. Aside from patients who previously underwent gastric banding, pregnant patients with prior MBS should not undergo GDM screening using any of the two aforementioned approaches. First, the diagnostic cut-off values used to define abnormal GCT and OGTT were determined in pregnant patients without prior history of MBS. Due to the altered anatomy and physiology following MBS, glucose kinetics are significantly modified, rendering these diagnostic criteria irrelevant in post-MBS pregnant patients. Furthermore, both GCT and OGTT are liquid meals, which pass quickly to the small intestine and may elicit a hyperinsulinemic hypoglycemic response, resulting in hypoglycemia in a substantial proportion of patients [56,60,61,62,63,64]. As such, alternative clinically relevant and safer methods are required. In our practice, we recommend post-MBS patients to undergo fasting glycemia and HbA1C analyses, and in the presence of overt diabetes or prediabetes, to plan the timing of pregnancy following thorough evaluation of diabetes-related complications and achieving optimal glycemic control. In the absence of pregestational dysglycemia, we repeat fasting glycemia (and HbA1C if not performed recently) analyses in order to identify early gestational diabetes mellitus based on the International Association of the Diabetes and Pregnancy Study Groups (IADPSG) criteria [65]. Unless dysglycemia has been identified, we repeat GDM screening between 24 and 28 weeks’ gestation by asking the patient to perform self-blood finger-stick glucose monitoring (SBGM), four to seven times a day, for 7–14 days, depending on the clinical characteristics. A more attractive approach, although not widely available in all countries, is the utilization of continuous glucose monitoring (CGM). CGM can provide valuable data on glucose kinetics and reveal the timing and frequency of hypoglycemia, thereby informing clinical management in post-MBS pregnant patients [59,66].

#### 4.1.4. Nutritional Follow-Up During Pregnancy

A main challenge in the management of post-MBS patients is the increased risk of nutritional deficiencies [67]. The physiologic changes that occur throughout gestation may further augment the risk of such deficiencies. Thus, among post-MBS patients who conceive after surgery, the combined effects of the surgery and pregnancy may increase the risk of micronutrient depletion [68]. These deficiencies may affect maternal and perinatal outcomes [68,69]. Therefore, dedicated nutritional follow-up is required during pregnancy to optimize maternal nutritional status during the prenatal course.

Anemia is commonly encountered in post-MBS pregnant patients [19,68,70,71], and may be the result of different nutritional deficiencies, including iron, folate, or vitamin B12 deficiency. Increased rates of anemia were reported in association with longer surgery-to-conception interval and following malabsorptive procedures [53,68]. As anemia is well known to associate with numerous adverse pregnancy outcomes [72,73], assessment of its specific causes and provision of proper treatment are advocated. In those with iron deficiency anemia, failure to respond to oral iron supplementation, and gastrointestinal tolerance, are common indications for the use of intravenous iron supplementation, which is considered safe beyond the first trimester of pregnancy [74]. In those in whom iron deficiency anemia is discovered during the late third trimester, intravenous iron supplementation may be preferred in order to optimize hemoglobin levels in a timely manner as a preparation for delivery.

#### 4.1.5. Dietary and Physical Habits

Maintaining optimal dietary and physical activity habits is vital in the long-term postoperative period after MBS. This may be even more important during pregnancy. Due to the lack of specific guidelines for post-MBS pregnant patients, recommendations for gestational weight gain should follow those of the National Academy of Medicine similar to the general population of patients [75]. Difficulties in maintaining regular dietary habits and fear of recurrent weight gain are commonly reported among post-MBS pregnant patients [14]. Moreover, only a minority of post-MBS patients perform regular physical activity throughout gestation [14]. These important aspects should not be overlooked, with proper guidance given during the antenatal course.

#### 4.1.6. Surgical Complications During Pregnancy

Surgical complications of MBS can occur during pregnancy. While the risk of complications may be increased in those who conceived early after surgery, surgical complications have been reported many years after surgery [76]. Surgical complications of MBS throughout gestation are potentially serious, and may lead to maternal death and fetal loss [76]. The most common diagnoses include internal hernia, bowel intussusception, intestinal obstruction, band slippage, volvulus and intestinal perforation [76,77]. Diagnosis and management of these cases may be highly challenging and should involve a multidisciplinary team including general surgeons, maternal fetal medicine specialists and anesthesiologists.

#### 4.1.7. The Effect of Pregnancy on Long-Term Outcomes

One of the most frequent questions asked by reproductive-aged patients undergoing MBS is whether pregnancy will negatively affect the long-term weight loss outcomes of the surgery. Reassuringly, several studies have shown that weight loss outcomes for those who conceived after surgery are comparable to those who did not become pregnant after surgery [78,79,80]. Moreover, even among those who conceived more than once after surgery, weight loss outcomes were similar [78]. These data, coupled with the beneficial effects of MBS on other pregnancy outcomes, should assist women in their decision making in regard to pregnancy after surgery.

#### 4.1.8. Psychosocial Support of Reproductive-Aged Women

It is well-evidenced that MBS is a time of immense change for patients, as they learn to live with a changed body, especially with a rapidly changing physical appearance, learning to eat differently and negotiating potential relationship dynamics [81]. People who have undergone MBS and have strong psychosocial support often have better outcomes than those who do not [82]. As stated previously, pregnancy can often be a desired outcome of MBS, and care must to taken to support mental wellbeing before, during and after pregnancy, as evidence shows women may experience mental wellbeing issues such as anxiety [83]. Healthcare professionals should also be aware of the stigma of obesity, and that the impact of stigma may pervade long after weight has been lost [84]. Additionally, there are societal judgments about MBS as an intervention, with many scrutinized or scorned for their choice of weight-loss interventions, and these issues may present themselves in clinical encounters [81]. Care should be taken to have supportive conversations where the patient can express their feelings openly.

Pregnancy after MBS offers opportunities for reproductive healthcare specialists to work collaboratively with MBS multidisciplinary teams to develop patient care frameworks and protocols to support women with MBS and their reproductive healthcare needs, while also providing adequate psychosocial support.

## 5. Limitations

This perspective reflects current knowledge and expert opinion regarding the implications of MBS on reproductive health; however, several potential caveats should be acknowledged. First, although the current perspective integrates findings from key studies and findings from our group, the majority of the recommendations are based on observational data, expert consensus, or clinical experience, rather than level 1 evidence. Further studies are warranted to better delineate the effects of MBS on reproductive health and improve the guidance provided. Moreover, the diversity of MBS procedures, patient populations introduces heterogenicity that may not be fully captured in this perspective. Finally, certain areas—including the effects of obesity management medications and newer surgical techniques, and long-term psychosocial outcomes—warrant further investigation.

## 6. Conclusions

This article provides important clinical guidance for the optimal management of reproductive-aged women undergoing MBS. As MBS becomes increasingly prevalent among women of childbearing age, understanding the implications of surgery on reproductive health is of paramount importance. It is critical to improve reproductive health counseling and contraception provision for patients undergoing MBS. This could be deeply beneficial in removing barriers to meeting patients’ reproductive desires and preferences through shared decision-making.

## Figures and Tables

**Table 1:** Summarizes the key topics that should be discussed as part of the pre-operative counselling.

All Topics and Counselling Should Be Evidence Based on Current Research, and Reviewed Regularly	
Topic	Rationale
Sexual activity and contraception	To ensure women are aware that fertility is potentially increased after MBS and that appropriate contraception, accounting for the mechanisms of MBS, are appropriately prescribed and monitored
Timing of future pregnancy	To ensure that women understand that nutritional status should be optimized prior to surgery To develop a strategy for nutritional monitoring as part of pre-pregnancy planning
Expected pregnancy outcomes	To ensure that women are aware of the possible complications associated with pregnancy following MBS
Effect of pregnancy on MBS-related outcomes	To ensure that women are aware of the effects of pregnancy on weight loss outcomes longer term
Multidisciplinary care plans	Work collaboratively with patient and wider healthcare professionals outside MBS team to harness the collective expertise for optimum patient care
